# Organ Dynamics and Hemodynamic of the Whole HH25 Avian Embryonic Heart, Revealed by Ultrasound Biomicroscopy, Boundary Tracking, and Flow Simulations

**DOI:** 10.1038/s41598-019-54061-w

**Published:** 2019-12-02

**Authors:** Sheldon Ho, Wei Xuan Chan, Nhan Phan-Thien, Choon Hwai Yap

**Affiliations:** 1 Department of Biomedical Engineering, National University of Singapore, Singapore, Singapore; 2 Department of Mechanical Engineering, National University of Singapore, Singapore, Singapore

**Keywords:** Morphogenesis, Biomedical engineering

## Abstract

Congenital heart malformations occur to substantial number of pregnancies. Studies showed that abnormal flow biomechanical environments could lead to malformations, making it important to understand the biomechanical environment of the developing heart. We performed 4D high-frequency ultrasound scans of chick embryonic hearts at HH25 to study the biomechanics of the whole heart (atria and ventricle). A novel and high-fidelity motion estimation technique, based on temporal motion model and non-rigid image registration algorithm, allowed automatic tracking of fluid-structure boundaries from scan images, and supported flow simulations. Results demonstrated that atrial appendages were the most contractile portion of the atria, having disproportionately high contribution to atrial blood pumping for its volume in the atria. However, the atria played a small role in blood pumping compared to the ventricle, as it had much lower ejection energy expenditure, and as the ventricle appeared to be able to draw inflow from the veins directly during late diastole. Spatially and temporally averaged wall shear stresses (WSS) for various cardiac structures were 0.062–0.068 Pa, but spatial-averaged WSS could be as high as 0.54 Pa in the RV. WSS was especially elevated at the atrial inlet, atrioventricular junction, regions near to the outflow tract, and at dividing lines between the left and right atrium and left and right side of the ventricle, where septation had begun and the lumen had narrowed. Elevated WSS could serve as biomechanics stimulation for proper growth and development.

## Introduction

Congenital heart disease affects 0.6–1.9% of newborns^1^ and is a leading cause of birth defect related deaths^2^. Experimental work in small animal embryos suggested that abnormal blood flow patterns and forces and the consequent abnormal mechanobiology could be responsible for congenital malformations^3–5^. Guided by the same principles, cardiologists performed interventions in human fetal hearts to modulate hemodynamics and rescue single ventricular gestational outcomes, and achieved moderate success^6,7^. Therefore, conducting studies to understand the biomechanical environment of the prenatal heart is important. For such studies, small animal embryos, including those of zebrafish^8^, chicken^9^ and rodents^10^, serve as excellent models to understand embryonic heart biomechanics as well as to gain insight into the interplay between the hemodynamic forces and resultant cardiac development.

In the developing heart, the atria is an important cardiac structure, but is not well studied. When its function was experimentally disrupted, cardiac malformation or maldevelopment resulted. For example, experiments knocking out atrial contractile mechanisms resulted in non-viable embryos^11^. Furthermore, embryos with ligation of the left atrium developed symptoms similar to the hypoplastic left heart syndrome^3^. Yet to date, the atrial function is not completely understood. For example, the atria have an appendage on each side, but the function of the appendages, if any, is currently not known.

We previously presented a technique to image the avian embryonic heart non-invasively in 4D using high-frequency ultrasound, and highlighted the subsequent image-processing necessary to distinguish between blood and tissue spaces^12,13^. We applied this technique to support image-based computational fluid dynamics (CFD) flow simulations of the embryonic common ventricle^12^ and aortic arches^13^ to understand the embryonic cardiac biomechanics. In the current work, we extended our imaging and simulations to the whole avian embryonic heart involving both the atria and the ventricle, to investigate atrial biomechanics, and the interplay between the two structures.

To aid the biomechanics study, we implemented a novel automatic cardiac motion estimation algorithm, via prescribing a cyclic motion mathematical model to 3D pair-wise non-rigid image registration, and adopted careful optimization to ensure accurate cardiac boundary motion tracking. This approach enabled the reduction of errors and uncertainties associated with manual segmentation. Interestingly, the motion tracking algorithm also enabled calculations of the surface deformational stretch.

## Method

### Imaging of chick embryos

All experimental work in this study were reviewed and approved by the Institutional Animal Use and Care Committee of the National University of Singapore. The experiments were conducted in accordance with guidelines and regulations. Fertilized Bovans Goldline chicken eggs were sourced from a local farm, and were incubated blunt end up, at 38 °C and approximately 60% humidity for 108 hours to obtain chick embryos at HH stage 25. Before imaging, the eggs shells were windowed from the top and shell membranes were removed for imaging, but could be resealed with Parafilm^®^ and returned to the incubator after scanning.

We used the same scanning technique previously reported^12,13^. A high frequency ultrasound system (Vevo2100, Visual Sonics Inc., Canada) with a MS700 transducer (30–70 MHz) was used. During scans, a sterilized polyurethane membrane was placed over the exposed embryo before warm ultrasound gel was placed on the membrane. This prevented direct contact of the embryo with the gel. The egg was mounted on a micro-adjustable stage and cine-images of the embryonic heart were taken at multiple planes spaced 50 μm apart. At each imaging plane, images of at least 25 cardiac cycles were taken at 50 frames per second. The temperature of the egg was maintained with a custom heat pad with thermostat control. During scans, the Doppler velocity of blood exiting the ventricle was captured and smoothed to remove noise.

Since there was no natural contrast between tissue and blood spaces in the high-frequency ultrasound images of embryos, the scans were processed with quadratic ensemble averaging algorithm guided by spatial and temporal image correlations, as described previously^12,13^, to create the required contrast. This algorithm tapped into the elevated temporal intensity dynamics of blood spaces to distinguish them from the tissue spaces, which had higher temporal intensity persistence.

### Non-rigid image-registration determination and tracking of cardiac boundaries

A novel image-processing technique was utilized to determine and track the cardiac boundaries to maximize the accuracy for calculating motion of all cardiac wall nodes for inputs into the computational fluid dynamics (CFD) simulations.

The segmentation of the cardiac myocardium-blood boundary at one initial time frame was first obtained, and nodes of this surface were automatically tracked for the rest of the cardiac cycle. Pairwise non-rigid image-registration of 3D images was performed using an open source module, Elastix^14^, to obtain displacements between neighbouring time points, using mutual information as the objective term and transform bending energy penalty as the regularization term. Next, the motions of nodes were modelled as a Fourier equation over time, and the Fourier coefficients were modelled as B-splines over space. An iteratively curve-fitting process was conducted in which the B-spline Fourier coefficients that had the best fit with the displacement fields from all time points were found. In the Elastix module, the objective-to-regularization term ratio was found to be optimal at 0.1. In the motion modelling, the B-spline grid spacing was found optimal at 0.1 mm. These settings should have sufficient resolution for the subject embryonic hearts, which were approximately 1.5 mm in size. Yet, they provided sufficient smooth boundaries without excessive creases.

The initial segmentation was performed with a simple thresholding algorithm using open-source software, VMTK (www.vmtk.org). The threshold intensity, however, was determined iteratively. At each threshold intensity, the image-tracking was performed as described above, and the volume changes in the ventricle were determined. When combined with subject-specific velocity measurements at the outflow tract, ultrasound could be used to calculate the velocity at the atrio-ventricular junction, based on an idealized parabolic flow profile assumption, and cross-sectional area measurements from that segmentation. The threshold intensity in which the flow velocities at the junction best matched literature measurements of normal chick embryos of the same stage was adopted^15^.

### Computational fluid dynamics simulations

The CFD simulations were run using Fluent Workbench (Ansys Inc., PA, USA). The wall movement was calculated with the boundary tracking algorithm described above, via a user-defined function. Each embryonic heart was meshed into a grid with at least 400,000 cells, as informed by a mesh convergence study. Mesh convergence study was conducted using the ventricle, in which the number of elements in the mesh was continually doubled until the difference in the average WSS between the final and preceding mesh was less than 1%. The mesh density in terms of number of elements per volume was then retained and used for all the simulations.

Simulations were conducted with time steps of 0.001 s, or 460–500 time steps per cardiac cycle. To remove artefacts due to the static fluid initial condition, simulation was continued until cyclic convergence was observed, in which the average WSS had less than 0.5% difference from that in the same cardiac phase of the previous cycle. Since the Reynolds number was low (<50), this typically occurred in less than 2 cardiac cycles. A Newtonian model was adopted for blood with a viscosity value of 0.0015 Pa.s^16^.

From the CFD results, the spatial and temporal characteristics of WSS were analysed, and the oscillatory shear index (OSI) was calculated to investigate the extent by which wall shear stresses were oscillatory^17^:

1 $$OSI=\frac{1}{2}(1-\frac{|{\int }_{0}^{T}\overrightarrow{\tau }\,dt|}{{\int }_{0}^{T}|\overrightarrow{\tau }|\,dt})$$

where *T* was the cardiac cycle duration, *t* was time, and $\overrightarrow{\tau }$ was the WSS vector. Ejection work done was calculated by combining our simulations results with measured absolute pressures from the literature^18^, as follows:

2 $${W}_{ejection}={\int }_{systole}{\int }_{surface}{P}_{wall}\,\ast \,({\boldsymbol{v}}\cdot \hat{{\boldsymbol{n}}})\,dA\,dt,$$

where P_wall_ was the pressure at the endocardial wall (the normal traction to the wall), calculated from the literature absolute pressures and simulation intraventricular pressure gradients, ***v*** was velocity, $\hat{{\boldsymbol{n}}}$ was normal, *A* was surface area of cardiac structure, and t was time.

The motions of the cardiac wall grid calculated by the boundary tracking algorithm was also used to compute wall deformational area stretch. This was done by taking the determinant of the deformation gradient tensor of the surface mesh.

### Simulation boundary conditions

The inlet to the atria was prescribed to have the reference pressure, and pressures everywhere else were calculated as the difference with this reference pressure. At the outlet, a 2-diameter long flow extension was modelled to counter boundary condition artefacts, at the end of which, uniform flow velocities were prescribed. An iterative approach was adopted to obtain the simulated velocities at the outlet to match that indicated by Doppler measurements, due to the difficulty in determining the outlet flow profile. The volumetric outflow rate was initialized with a parabolic flow profile that matched Doppler measurements. Subsequently, the CFD simulation was iteratively repeated so as to update the flow profile. This was done until a satisfactory match between CFD and Doppler velocities was obtained.

## Result

### Image threshold identification

**Figure 1:**
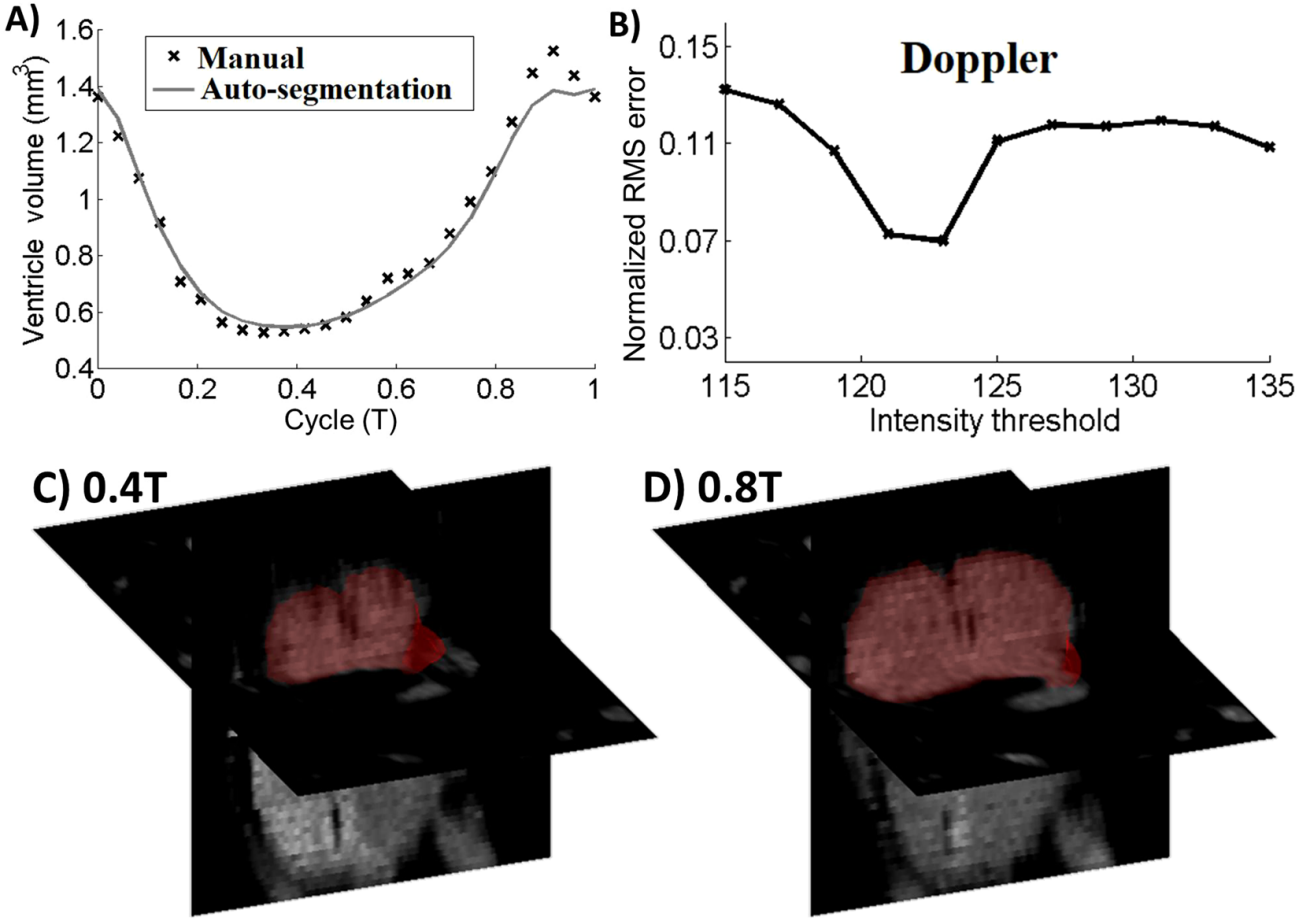
(**A**) Volume of the embryonic ventricle obtained from automatic cardiac motion estimation and from manual segmentation at every time point, demonstrating a good match. (**B**) Normalized RMS error when comparing atrioventricular junction flow velocities calculated with automatic algorithm with that obtained from literature^15^. Adjusting the image intensity of segmentation enabled minimization of this error. (**C,D**) The segmentation of the heart in red had qualitative good fit with boundaries of blood space on the ensemble averaged ultrasound images, at two time points in the cardiac cycle (T = duration of cycle).

The initial segmentation of the cardiac blood space and the tracking of the blood-tissue boundary depended largely on the threshold intensity value used during segmentation, and could vary significantly with different threshold. This created uncertainty as to which threshold would be realistic. Optimization was done by evaluating results at various threshold intensities and comparing the flow velocities at the atrioventricular junction consequent to each threshold, with flow velocities obtained from literature values (Fig. 1B). In this chick embryonic sample, the intensity value of 124 was found to be optimal, and was used for subsequent analysis.

After this optimization, the segmentation obtained from the automatic cardiac motion estimation compared well with the segmentations at every time point using the VMTK software. This was demonstrated by the volume of segmented ventricle versus time plot in Fig. 1A. Sample images comparing the image and the segmented boundaries demonstrated satisfactory fit, as shown in Fig. 1C,D.

### Embryonic heart anatomy and function

**Figure 2:**
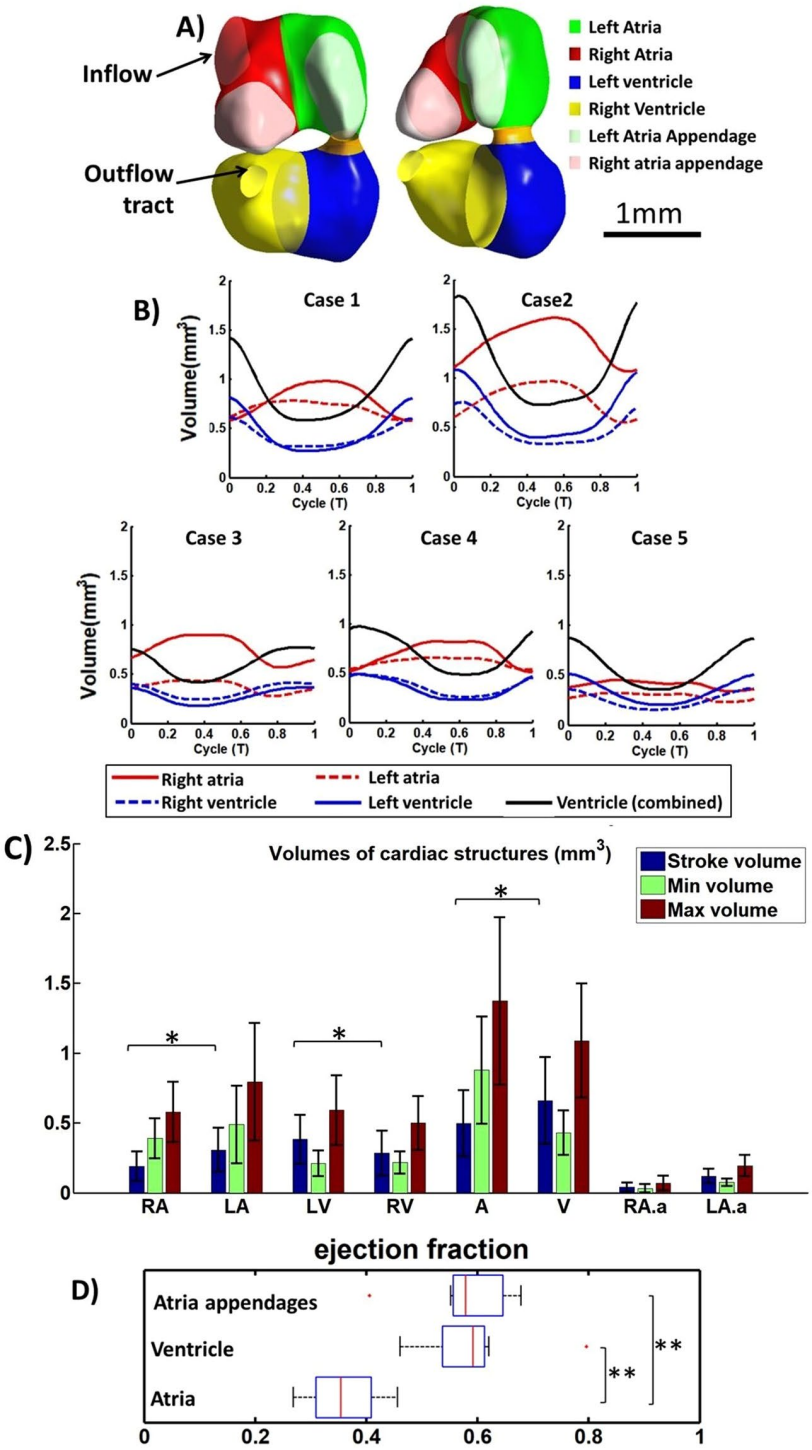
(**A**) The embryonic heart, divided into the various cardiac structures, with ventral and side view. (**B**) Volume waveforms of different cardiac structures over a cardiac cycle for 5 specimens. (**C**) Maximum and minimum volumes of different cardiac chambers, and their stroke volumes (n = 8). (**D**) Ejection fraction of different cardiac structures (n = 8). Red line - median, box edges – 25th and 75th percentile, whiskers – maximum/minimum excluding outliers, red dots – outliers (defined as outside 1.5 times the interquartile range). The atrial appendage played out-sized role in blood pumping, contributing 32.3 ± 7.0% of atria stroke volume despite only occupying 20.1 ± 5.6% of atrial end-diastolic volume. RA- Right Atria, LA-Left Atria, RV-Right Ventricle, LV-Left ventricle, A-Combined left and right atria, V-Entire primitive ventricle, RA.a-Right atria appendage, LA.a-Left atria appendage. *p < 0.05, **p < 0.0001.

The embryo was imaged at 4.5 days old, just prior to septation, so as to provide a better understanding of hemodynamic environment which would lead to septation. At this stage, the embryonic heart had a very different geometry than adult heart, having two atria that are joined, and one single ventricle which had yet to separate into their left and right chambers. However, the ventricle showed sign of septation as evident by a narrower lumen around its middle. The atrial appendages could also be prominently observed as protrusions at the anterior face of the atria, indicated by a lighter shade of the atrial color in Fig. 2A. Ventricular contraction occurred rapidly during systole, and the ventricle then stayed contracted for a transient period before gradually relaxing over a longer time period. Conversely, the relaxation of the atria quickly occurred following its contraction, and then the atria maintained its relaxed state for a period before the next contraction. Atrial contraction occurred directly in synchrony with ventricular relaxation and vice versa, and there were no division into E-wave and A-wave motions, as would be observed in fetal^19^ and adult hearts^20^.

Figure 2 also illustrated the details of cardiac structure volumes over time for 5 embryos, and reported cardiac structure volumes and stroke volumes for 8 embryos. The average stroke volumes for the embryos were 0.50 ± 0.24 mm^3^ and 0.66 ± 0.31 mm^3^ for the atria and the ventricle respectively. Statistical analysis demonstrated that this lower stroke volume of the atria compared to the ventricle was significant (p = 0.0183), corroborating with earlier observations^12^. This suggested that some inflow into the ventricle came directly from the veins and passed through the atria without being propelled by atrial contractions. The mean of the end-diastolic volume was 1.37 mm^3^ for the atria and 1.09 mm^3^ for the ventricle, at their respective end-diastolic times. The ejection fraction of the atria was 0.36 ± 0.07 which was significantly lower (p = 0.00004) than the ventricle’s ejection fraction of 0.59 ± 0.10 (Fig. 2D). An interesting observation was that within the atria, the appendages underwent significant contraction every cardiac cycle, and were significant contributors to the atria’s ejection fraction. Their overall ejection fraction was 0.58 ± 0.09, which was similar to that of the ventricle, and much higher than that of the atria (p = 0.00008). To further demonstrate this, the appendages occupied only about 20.1 ± 5.6% of the atria’s end-diastolic volume but contributed about 32.3 ± 7.0% to the stroke volume, and there was a statistically significant difference between these two percentage data (p = 0.0001). A further interesting observation was that the left atrium and left side of the ventricle appeared to have significantly higher stroke volumes compared to the corresponding right side (p = 0.0176 for atria and 0.0302 for ventricle).

**Figure 3:**
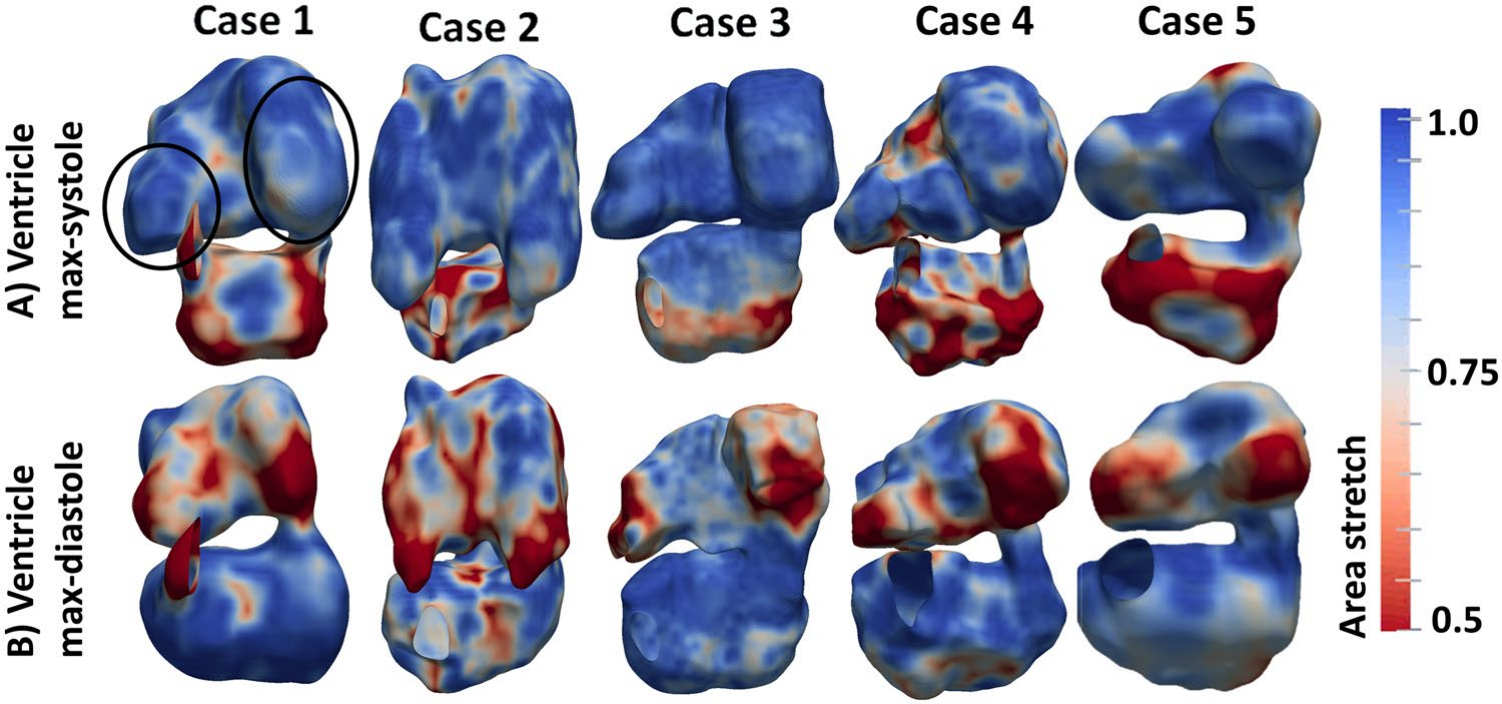
Area stretch ratio of the cardiac walls at (**A**) mid-ventricular-systole and (**B**) mid-ventricular diastole for the 5 embryonic subjects evaluated. Circled area showed the appendages. Area stretch of 1.0 indicated no contractile stretch, while area stretch of less than 1.0 indicates contraction. A video of area stretch for case 2 was uploaded as online Supplementary Video 1.

Deformational area stretch ratio of the myocardial wall (current wall area/initial wall area) could be calculated from the spatial motion of cardiac wall points obtained from the image-tracking algorithm, with the maximally stretched state of any mesh element taken as the zero stretch reference. Results were shown in Fig. 3. The peak area compression, averaged across all ventricular walls in 3D, was 43 ± 9% for our five subjects. This was higher but comparable to 27% reported in a previous study^21^ (converted from strain components to area stretch), which quantified strain with 2D microscopy at a slightly earlier stage (HH24 instead of HH25). Interestingly, high cumulative wall stretches and stretch rates were found to be concentrated at the atrial appendages during atrial systole (ventricular diastole), and were distributed across the ventricle during ventricular systole (atrial diastole). This demonstrated the highly contractile nature of the atrial appendages, and corroborated with the stroke volume measurements above.

### Flow fields in the embryonic heart

**Figure 4:**
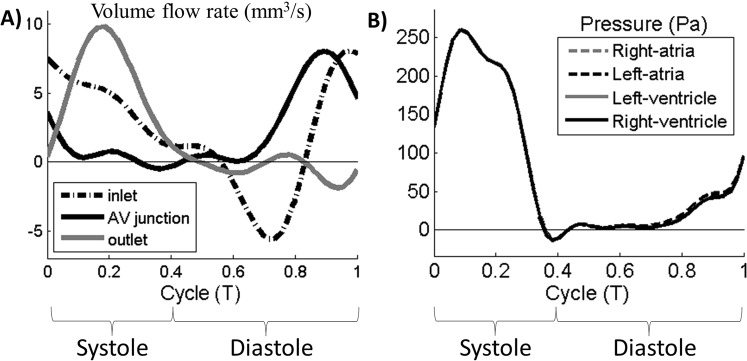
(**A**) Volume flow waveform in the embryonic heart at the atrial inlet, the ventricular outlet and the atrioventricular (AV) junction. (**B**) Pressure waveform in the different cardiac structures. Pressure waveform value was taken from literature and applied at the ventricle outlet^15^. Pressure difference from flow simulations was then used to compute pressures in other parts of the heart. Systole and diastole in this figure refered to ventricle systole and ventricle diastole respectively.

The volumetric flow rate at the atrial inlet, ventricular outlet and atrioventricular junction were shown in Fig. 4A. The flow waveform at outlet was based on our Doppler measurements and was observed to be similar to previous studies^12,22^. Our optimization attempts led to a successful match between the flow rate at the atrioventricular junction and the measurements in the literature^15^. At the atrial inlet, substantial reversed flow was observed at the onset of atrial systole. However, during late atrial systole, despite atrial contractions, inflow occurred at the atrial inlet instead of blood being pushed out of the inlet as reversed flow. This atrial inflow was drawn by the relaxing ventricle, and will be further demonstrated in the sections below. In Fig. 4B, the ventricular pressure waveform was obtained from the literature^18^ and applied at ventricular outlet. Pressure difference across the different cardiac structures was small relative to the magnitude of the pressure waveform. During ventricular systole, however, when the atrioventricular junction was collapsed^23^, the atria were de-coupled from the ventricle, and no pressure data for the atria was available for this period.

Due to low Reynolds number of less than 50, flow in the embryonic heart was observed to be laminar with no sign of flow separation despite large angles of turning at certain locations. Moreover, there was an absence of chaotic mixing despite the presence of converging and splitting flow streams. The highest flow velocities occurred at the atrial inlet, the atrioventricular junction, and at the outflow tract, all at different timings during the cardiac cycle. Velocities were high in these channels as they were the narrowest. Due to the lack of valves between the various structures, velocities at these junctions and boundaries exhibited significant oscillations.

**Figure 5:**
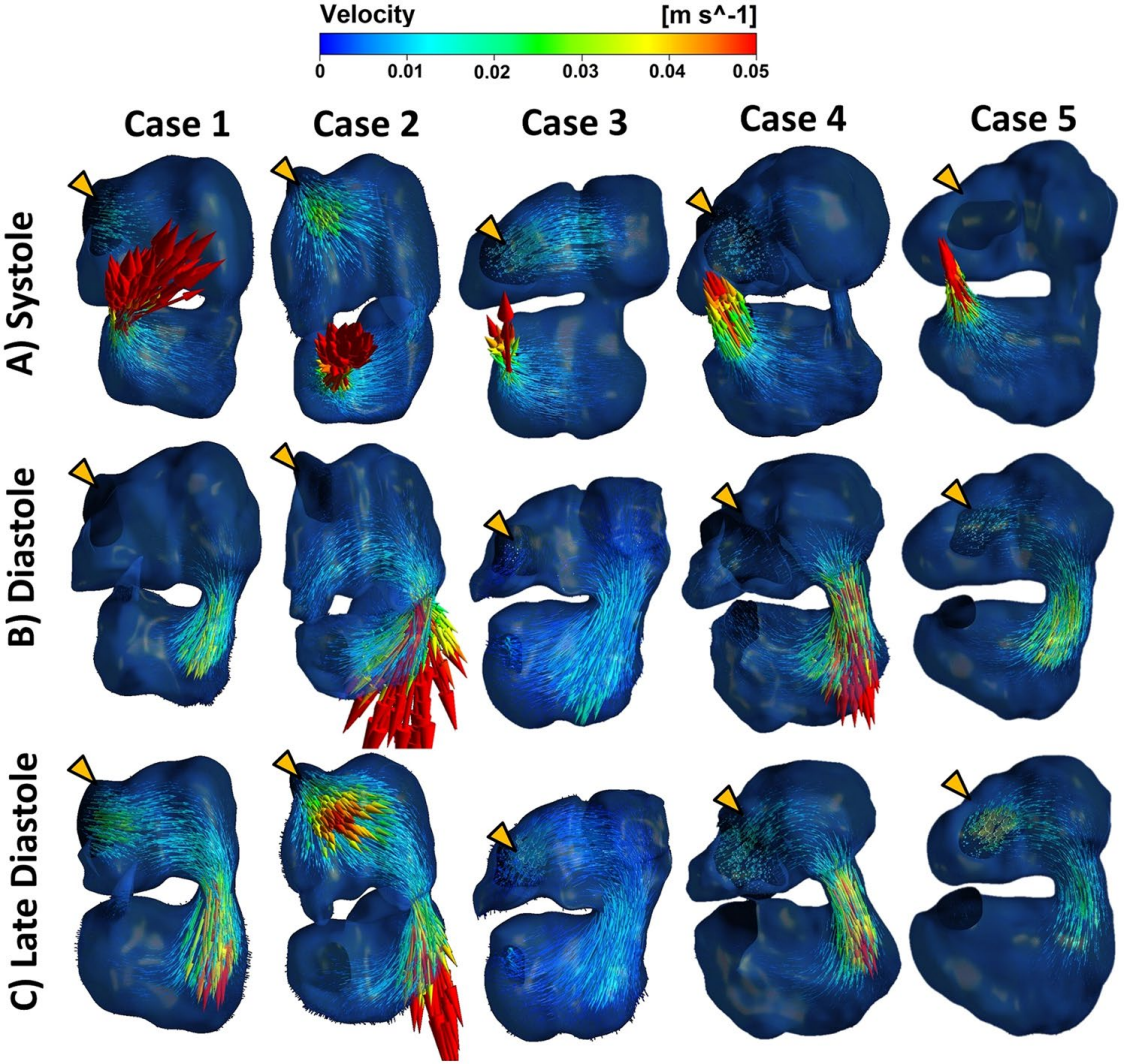
(**A**) The ventricle contracted during ventricle systole, pumping blood into the outflow tract. At the same time, atria relaxed, causing inflow from the veins. Contraction of the atrioventricular canal prevented backflow of blood from ventricle to atria. (**B**) Ventricular filling phase whereby the contracting atria pumped blood into the ventricle. (**C**) After atria contraction, the ventricle expanded further, which caused blood to flow directly from Sinus Venosus towards the ventricle. Systole and diastole labels in this figure referred to those of the ventricle. Orange arrows indicated position of venous inlet, which was behind the embryonic heart. Videos of flow fields were uploaded as Supplementary Videos 2–6.

During ventricular diastole, a strong inflow jet could be observed through the atrioventricular junction, but velocity vectors could be observed towards both the left and right side of the ventricle, as both structures were relaxing and filling with blood (Fig. 5B). Nearing the end of ventricular diastole, ventricular expansion persisted even after atrial contraction ended, leading to continued inflow at the atrial inlet, which were directed toward the ventricle (Fig. 5C). Immediately after this diastolic phase, ventricular systolic contraction resulted in a strong forward flow out to the outflow tract with well-aligned velocity vectors.

Atrial flow fields were slightly more complex, and subtle features could best be observed from Supplementary Videos 2–6. During atrial diastole, atrial inflow velocity vectors pointed towards atrial appendages and as well as other parts of the atria (Fig. 5A). During atrial systole, atrial contraction and ventricular relaxation worked in concert to cause blood to move in high velocities through the atrioventricular junction into the ventricle, and to generate high wall shear stresses at the junction (Fig. 5B). During initial atrial systole, a transient reversed flow was observed at the atrial inlet as the atria pushed some blood backwards into the veins. However, this was quickly followed by forward inflow at this inlet despite the continued contraction of the atria, most likely because the expanding ventricle created a suction to pull atrial and venous blood towards it (Fig. 5C). A suction effect could be created in the ventricle during ventricular diastole because the outflow tract underwent luminal collapse and adopted a valve-like function^24^, resulting in ventricular expansion pulling fluid in only through the veins, and not from the outflow tract. In fact, the inflow at the atrial inlet under the influence of this ventricular diastolic suction has comparable velocities as that during atrial diastole. Furthermore, since the atrial appendages had significant contraction, they generated velocity vectors that converged with venous inflow vectors.

**Figure 6:**
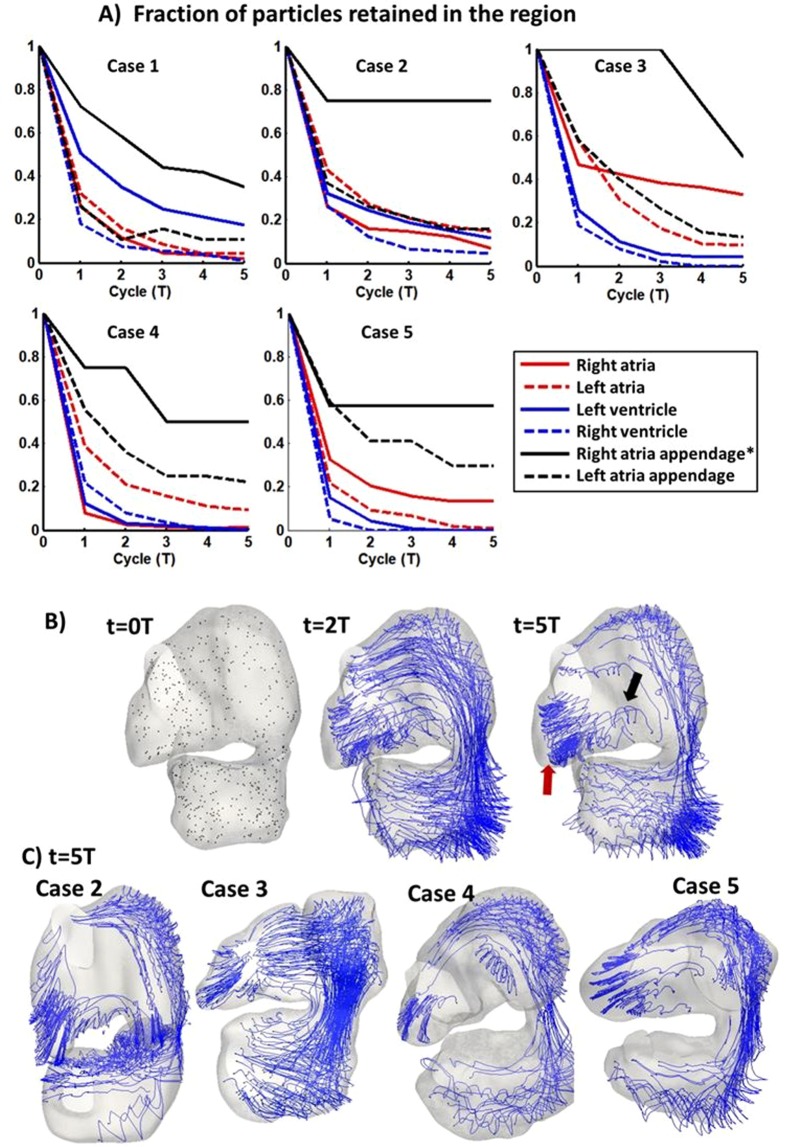
(**A**) Plots demonstrating blood particle retention in various cardiac structures, quantified as the fraction of particles retained in the structure, after an initial random seeding and subsequent tracking for 5 cardiac cycles. Particles in right atrial appendage were found to have higher retention rate. Right atrial appendage exhibited significantly higher particle retention than other region (*p < 0.005). (**B**) Particles and path lines of remaining particles in entire heart (embryo #1) after a few cardiac cycles, (**C**) particles and path lines of remaining particles after 5 cycles for subject #2–5. Red arrow: particles oscillating within the right atrial appendage. Black arrow: there was only minimal migration of particles out of the right atrial appendage. A video of the particle tracking for case 1 was added as Supplementary Video 7.

To visualize flow pathlines and to investigate blood retention time in the various cardiac structures, 500 particles were seeded randomly across the entire embryonic heart volume and tracked over 5 heart beats. These results were shown in Fig. 6. An interesting observation was that blood in the right atrial appendage had a very long retention time, with a mean particle retention fraction of 58 ± 19% even after 5 cycles. Path line visualization demonstrated that particles in the right atrial appendage mostly moved in an approximately linear and oscillatory pattern over the cardiac cycle. Only occasionally would particles further away from the appendage apex drift out and be washed away. Blood particle retention ratio of the right atrial appendage and other cardiac structures were significantly lower (p < 0.005).

This high retention time in the right atrial appendage was most likely a consequent of the appendage geometry, which was like an elongated side branch from the atria that could provide shelter to blood from washout forces, and that the appendage was far away from the high velocity streams in the atria. The same high retention time was not shared by the left atrial appendage as it was generally not as elongated. Also, the left appendage was located close to the atrioventricular junction where very high flow velocities were periodically manifested to wash out particles.

Another interesting observation was that there was higher blood particle retention along the left free wall of both the left atrium and the left ventricle, where particles bobbed around with cardiac contraction motions with low mean forward flow velocities. This was likely the result of these locations being further away from the high velocity streams. Thus, these cardiac structures on the left often had higher retention time compared to those on the right. The cardiac structure with the lowest retention time was the right ventricle as its entire luminal space was exposed to high velocity blood outflow, and there was no sheltered pocket for blood retention.

### Blood flow wall shear stresses

**Figure 7:**
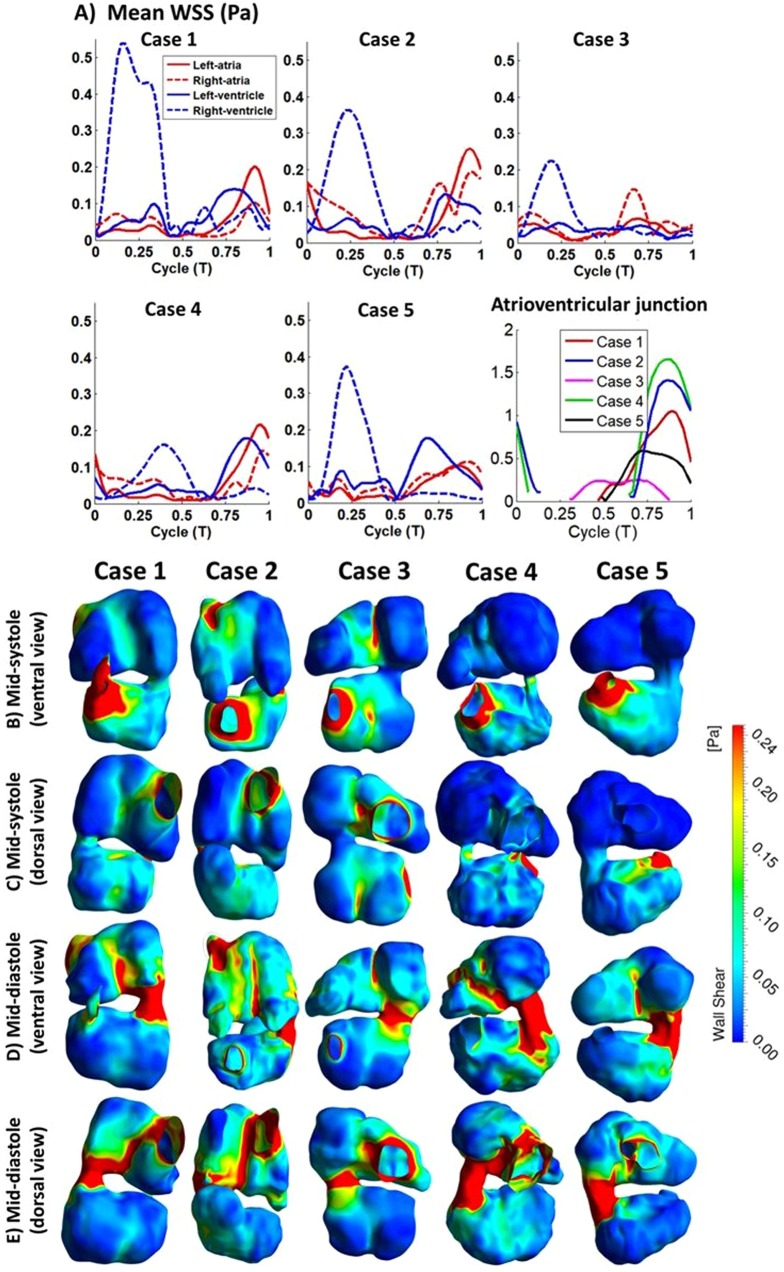
(**A**) Averaged WSS magnitude of different heart structures across a cardiac cycle and at atrioventricular junction for 5 embryos. (**B–E**) Surface WSS plot of embryonic heart. Higher WSS were noticed at region with narrow area such as the inlet, atrioventricular junction, outlet, middle region of atria, and mid region of ventricle. (**B,C**) Ventricle mid-systole, maximum flow at ventricle outlet. (**D,E**) Ventricle mid-diastole, maximum flow at ventricle inlet (**C,E**) dorsal view. (**B,D**) ventral view.

Since blood flow WSS was believed to be a mechanobiological stimulus that was relevant to embryonic cardiovascular development, WSS was calculated from flow simulation results and investigated. Results were shown in Fig. 7. Generally, flow near to cardiac walls was often dynamic; changing directions quickly and often. This occurred due to the pulsatile movements and complex geometry of the heart

There were significant spatial and temporal variations of WSS magnitudes. A few regions experienced high magnitudes of WSS, such as the right ventricular wall close to the outflow tract, the atrioventricular junction, and regions in the right atrium near to its inlet. High WSS was found here due to high flow velocities and narrow luminal channels. The dividing lines between the two atria and between the two sides of the ventricle were also locations of high WSS. At these locations, septation growth resulted in protrusions into the luminal space, which led to narrower flow channels and higher velocities and thus higher WSS. Conversely, at regions which recessed away from the lumen, such as atrial appendages, the left free wall of the left atrium, and posterior free walls of the ventricle away from the midline, WSS was significantly lower.

Plots in Fig. 7 demonstrated the spatial variations of the average WSS within each cardiac structure. The highest WSS was found in the RV and occurred during ventricular systole, which was generated by blood ejection into the outflow tract. During systole, RV WSS could be up to 1–3 times higher than that of the LV, consistent with our previous report^12^. As previously explained, this was because the stroke volume of the LV needed to pass through the RV before being ejected, and thus the RV needed to accommodate higher flow rates than the LV. However, LV WSS could also elevate during ventricular diastole due to the manifestation of the atrioventricular junction fluid jet in the LV. In some cases, such as embryo #4, this could lead to significant magnitudes, due to a narrow atrioventricular junction that generated high inflow jet velocities. As for the atria, the WSS magnitude was similar to the LV, and was similarly elevated at two time periods, during atrial diastole when inflow occurred, and during atrial systole when ejection occurred. An outlier that was observed was for embryo #3, where the atrioventricular WSS was lower than the other samples. This could be a result of natural variability, or a slight difference in the developmental stage of these subjects, being a few hours younger.

**Figure 8:**
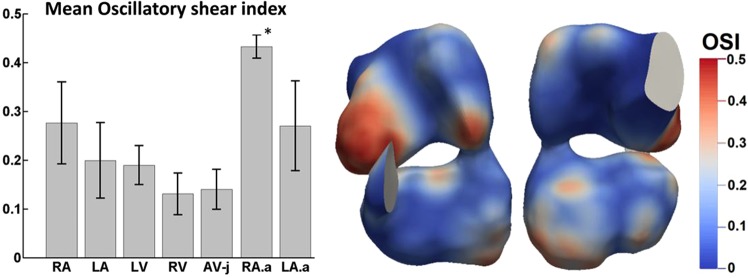
(left) Oscillatory shear index (OSI) of various cardiac zones (n = 5). The right atria appendage exhibited significantly higher OSI than other region (*P < 0.006 compared to all other cardiac zones). (right) Spatial variability of OSI. RA, LA - Right Atria and Left Atria inclusive of their appendages, LV-Left ventricle, RV-Right Ventricle, AV-j-Atrioventricular junction, RA.a-Right atria appendage, LA.a-Left atria appendage.

Due to the complex and dynamic flow patterns, WSS in the embryonic heart had significant oscillatory characteristics, as could be observed in significant OSI values (Fig. 8). The highest OSI (0.43 ± 0.02) was found in the right atrial appendage, consequent to the oscillatory flow pattern as discussed in the previous section, which was significantly higher than any other cardiac structure (p < 0.006). In the left atrial appendage, statistical significance was only found compared to left atrium and right ventricle (p < 0.05). Other regions where OSI were high included the left free wall of the LA and LV, corresponding to locations where particle retention was higher.

### Work done and pressure-volume (PV) loops in embryonic heart

**Figure 9:**
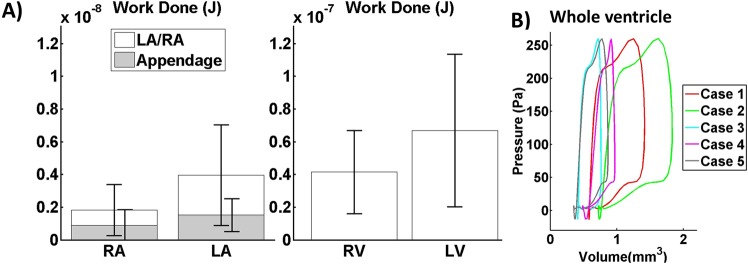
(**A**) Work done by the cardiac structures during contraction (n = 5). The ventricle performed much higher work than the atria and its appendages. The left side of the embryonic perform higher work due to greater motion and stroke volumes. Atrial appendages account for a large percentage of work done by the atria. (**B**) Pressure-volume loop of the whole ventricle. Work done and pressure volume loop were estimated using pressure waveform from literature^15^. RA- Right Atrium, LA-Left Atrium, LV-Left ventricle, RV-Right Ventricle.

The fluid ejection work done as computed with our CFD simulations were shown in Fig. 9A and the whole ventricular PV loops using volumes obtained in our study and pressures from Keller *et al*.^15^ are given in Fig. 9B. Generally, our HH25 PV loops adopted similar shapes as the HH24 pressure-area loops from Keller *et al*., but Keller *et al*. imaged in 2D and only reported 2D ventricular areas. Stekelenburg-de Vos *et al*. performed 2D imaging as well, but estimated 3D volume values by assuming an ellipsoidal ventricular shape^25^. For the HH24 chick embryo, they reported higher pressures than Keller *et al*., but had similar stroke volumes and slightly smaller diastolic volumes compared to our HH25 embryos (Stekelenburg-de Vos vs. our data: stroke volume of 0.750 mm^3^ vs 0.661 ± 0.307 mm^3^, and diastolic volume of 0.947 vs. 1.173 ± 0.446 mm^3^).

Generally, the left side of the heart had greater work done, due to generally greater motion and stroke volumes. The work done by the atria were found to be one order of magnitude lower (p = 0.0134) than that of the ventricle, even though both had stroke volumes of the same order of magnitude. This was because the ventricle contracted against high pressure and needed to expend much energy to counter the pressure, but the atria did not. This suggested that the atria had low energy contribution towards circulation. Within the atria, the appendages were found to have significant contributions towards atrial ejection work done (42.5 ± 6.0%), which was significantly larger than its size ratio within the atria (20.1 ± 5.6%) with p = 0.0123.

## Discussion

The current study is the first 4D flow simulation of the whole atrio-ventricular embryonic heart based on *in vivo* scans of cardiac motions, and careful image analysis. To achieve this, we employed a recently developed imaging technique that could generate blood-tissue contrast from high frequency ultrasound images of embryos^12,13^, which enable 4D imaging of embryos with sufficient resolution for detailed biomechanics and cardiac function studies, and which gave sufficient imaging depth to capture the entire heart. Further, we employed a novel automated technique to track the tissue-blood boundary that combined 3D pair-wise image registration with a cyclic and smooth mathematical model of wall motions, and had to take much care to optimize segmentation thresholding and curve-fitting settings for accuracy.

This approach enabled good 3D estimates of cardiac structure volumes and stroke volumes, which were previously done in 2D manner^26^, or calculated from 2D scans based on idealized geometric assumptions^27^. The surface tracking algorithm reduced the time needed for segmentation of the heart’s geometry, as segmentations did not need to be performed at every time point. It also opened up new capabilities, such as enabling the estimations of myocardium stretch for investigations of localized contractility, and allowing a more realistic description of the cardiac wall motions compared to our previous implementation^12^, and thus providing more realistic boundary conditions for our 4D simulation of flow. In the past, a majority of embryonic heart flow simulations were performed in 2D^28^ or in the quasi-static form^22^. Recently, however, there is a gradual trend towards 4D imaging and simulations to achieve greater accuracy and realness^12,29^.

Our results revealed interesting physiological function of the atria. At HH25, the atria had yet to septate, but the atrial appendages were already present. The embryonic atrial appendages were found have higher contractility than other parts of the atria, and were comparable to ventricular myocardium. Although occupying only ~20% of the atrial diastolic volume, they accounted for ~32% of the stroke volume, and ~42% of the work done by the atria, and they had the highest ejection fraction compared to any other cardiac structures. This suggested that the atrial appendages might have the function of enhancing atrial blood pumping. The anatomy of the appendages was that of elongated structures branching out from the main atrial chambers, and they generated stroke volumes mainly via shortening. This seemed to be a good way to generate stroke volumes without requiring drastic changes of the atrial dimensions, a notion that corroborated with the above hypothesis of their function.

To date, the function of atrial appendages is not understood, neither in adult hearts nor prenatal hearts. In adult hearts, the appendages have no apparent function, but are frequently sites for thrombosis, such as in 91% of patients with non-vulvar atrial fibrillation^30^. Various authors suggested pressure sensing and endocrinal roles for the appendage, but conceded that in cases where surgical ligation or ablation were performed on the atrial to reduce thrombosis and stroke risks, no deleterious effects were observed^31^. The atrial appendages in the embryonic heart looked similar to those in adult hearts, but studies had found that the adult left atria appendages were formed from the entire primitive left atria, instead of an enlargement of the embryonic atria appendages^31^. Little is known about their function, and thus it was interesting that our results suggested a fluid pumping role for them.

However, our calculations of the ejection work output demonstrated that the atria energy output was one order of magnitude lower than that of the ventricle, suggesting that the fluid pumping function of the atria and its appendages were not essential for generating overall cardiac output. Further, our results suggested that the embryonic ventricles were capable of directly drawing blood from the veins, as they naturally do during late atrial systolic phase. We thus hypothesize that the fluid pumping function of the atria might be important not for cardiac output generation, but for generation of flow forces stimuli for the proper heart development. A previous study knocking out the contractile function of the atria found that this led to fetal non-viability, as the flow abnormalities had led to atrioventricular endocardial cushions defects, leading to septation and valve development problems^11^, corroborating with our hypothesis. In another study, atrial clipping in chick embryos was shown to lead to compensatory ventricular myocardial remodeling^32^, again demonstrating atrial pumping’s influence on the ventricle, most likely via hemodynamics.

It is thus important to characterize the flow patterns and hemodynamic forces imposed by blood on the cardiac structures, and to understand how abnormal patterns and forces can lead to malformations. It is generally understood that the embryonic endothelium can respond to flow WSS and provide signals for myocardium and cushions development to influence remodeling and growth^9,33,34^, and that endothelial cells can differentiate between oscillatory shear stresses from persistent ones^35^. In particular, a study disrupting normal atrioventricular junction flow via rapid pacing of the chick embryonic heart to cause excessive regurgitation led to the observation of atrioventricular cushions defects^36^. These highlight the importance of understanding the details of the embryonic heart WSS.

For this reason, we studied and reported the embryonic heart WSS and their oscillatory characteristics in this study. Generally, the WSS observed had substantial oscillatory nature, due to flow complexity introduced by the heart motions. For comparison purposes, we performed estimated the OSI in other cardiovascular structures, using plots from literature and simulation data from our previous work. We estimated OSI to be 0.04–0.2 in human adult aorta^37^, 0.22–0.31 in human adult ventricles^38,39^, about 0.17 in human fetal right ventricles^40,41^, and 0.1–0.3 in zebrafish ventricles^29^. OSI in our embryonic hearts thus had the same ranges as those observed in hearts of all scales, including human adult and fetal, and zebrafish hearts, but were higher than vascular structures, where flow tended to align in the stream-wise direction and OSI were low. Oscillatory characteristics were especially high in the right atria and appendage with high OSI. In terms of WSS magnitudes, they were especially elevated at the atrioventricular junction, reaching magnitudes of 0.26–1.4 Pa. Elevations were also observed at the developing septum, as the narrowed down the heart lumen, and caused higher velocity in passing flow, reaching magnitudes of 0.13–0.16 Pa. These high shear stresses could be important stimuli for normal septation and valve development.

In our study, among the 5 embryonic subjects studied, there were substantial variability in the shapes and size of the hearts and their chambers. These could be due to natural biological inter-subject variability, but it could also be due to differences in developmental stage at the time of the scan. For example, embryo #3 had the smallest cardiac chamber sizes, stroke volumes, flow rates, and WSS magnitudes, which could be due to the embryo developing slower than its counterparts or being a few hours younger. We noted, however, that despite the variability, the observations discussed above could be uniformly observed for all the embryos, such as their ejection fractions, flow patterns, oscillatory shear indices, ejection energy measures, particle retention in the atrial appendages, and the relatively high contributions of the atrial appendages in fluid pumping.

In our studies, the atrial volume waveforms were found to have shapes that were skewed towards the later time, bearing resemblance to previous measurements performed in 2D by Campbell *et al*.^26^. The third and fourth embryos’ atrial volume waveform had a flatter peak, which resembled that measured for younger embryos in Campbell *et al*. This could indicate that these embryos were developmentally younger. Further, analysis of the flow waveforms and volumetric changes of the ventricle revealed no distinct E- and A-wave, which matched with ultrasound Doppler measurements at the atrioventricular junction^23^.

## Limitation

The primary limitation of the current study was the inability of the imaging method and the dynamic mesh simulation technique to model the collapse of the atrioventricular junction during ventricular systole, which was reported by previous investigators^23^. However, in our model, there was little flow through the junction during ventricular systole, thus retaining resemblance to actual hemodynamic conditions. In view of this, we disregarded the ventricular systolic WSS results at the atrioventricular junction. Secondly, our image segmentation and registration disregarded myocardial trabeculations, as we focused on obtaining the larger scale flow features, rather than investigating localized micro-flow features. This could have resulted in changes to volumetric parameters, flow patterns and wall shear stress results. Finally, blood was modelled as a continuum rather than particulate fluid, but this should be sufficiently accurate, as blood cells were small compared to the embryonic heart size scale.

## Conclusion

We performed the first whole heart (atria and ventricle) image-based simulations of the embryonic heart, using HH25 chick embryos as subjects. We utilized 4D high-frequency ultrasound imaging of the embryonic heart, and adopted a novel image-registration and a cyclic mathematical model to track myocardial motions, which enabled computations of myocardial stretches and enabled 4D flow simulations. Results demonstrated that the atria only had minor blood pumping energy contribution, and that the ventricle was capable of drawing blood directly from the veins. We thus hypothesize that the mechanical function of the atria were not to generate circulatory energy, but to create flow forces stimuli for proper development. Within the atria, the appendages had out-sized contributions towards fluid pumping, which could be their function in the embryonic heart. Wall shear stress was also characterized, was found to be oscillatory in the right atria, the atrial appendages and the left ventricle, and was found to be elevated at the atrial inlet, the atrioventricular junction, the outflow tract, and septation structures. These shear stimuli could be important to development.

## Supplementary information

Supplementary Video 1

Supplementary Video 2

Supplementary Video 3

Supplementary Video 4

Supplementary Video 5

Supplementary Video 6

Supplementary Video 7

## Data Availability

The datasets generated during and/or analyzed during the current study are available from the corresponding author on reasonable request.
